# Seat Belt Injury and Efferent Loop Laceration Following a Single Anastomosis Duodenal Switch: A Case Report

**DOI:** 10.7759/cureus.61388

**Published:** 2024-05-30

**Authors:** Alex Petrak, Emily A McNabb, Benjamin P Nguyen, Vincent E Kirkpatrick, Sebastiano Cassaro

**Affiliations:** 1 Department of Surgery, Kaweah Health Medical Center, Visalia, USA

**Keywords:** obesity management, blunt abdominal trauma, small bowel injury, seat belt injury, single-anastomosis duodenal switch

## Abstract

Blunt abdominal trauma patients who have had prior bariatric procedures may present a diagnostic and therapeutic challenge. The single anastomosis duodenal-ileal bypass with sleeve (SADI-S) is a modified duodenal switch procedure that is relatively uncommon. This case report describes a patient who previously underwent a SADI-S for the management of obesity and subsequently sustained a seat belt injury in a motor vehicle collision resulting in a laceration of the efferent loop. The patient presented with symptoms of acute abdominal pain and was diagnosed through imaging studies. Prompt surgical intervention was performed with resection and primary anastomosis of the damaged section of the jejunum, and repair of a large mesenteric laceration. We discuss the importance of early recognition and intraoperative decision-making in the case of this patient concerning her SADI-S.

## Introduction

In 2018, over 160,000 obese adults underwent a bariatric procedure in the United States [1]. The duodenal switch comprises approximately 1% of these [2]. A simplified version of this operation, the single anastomosis duodenal-ileal bypass with sleeve (SADI-S), was first described in 2007 [3]. It involves a sleeve gastrectomy followed by a single anastomosis between the duodenum and the ileum, combining restriction and malabsorption. SADI-S and the traditional double-anastomosis duodenal switch are comparable in terms of weight loss and complication rate [4,5].

Overall, seatbelt use has lowered major injury rates and improved mortality rates in motor vehicle collisions [6,7]; however, in a minority of patients, seatbelts have been shown to cause severe injuries to the gastrointestinal tract, vascular structures, and the spine [8,9].

We present a case of a 39-year-old female with a previous SADI-S who experienced a small bowel laceration following a seatbelt injury. To our knowledge, this is the first case report to discuss a seatbelt injury to a patient with a prior SADI-S procedure.

## Case presentation

A 39-year-old female with a history of morbid obesity and SADI-S two years prior was brought by ambulance to the emergency department for trauma evaluation after the car she was driving collided head-on with a larger vehicle at highway speeds. The patient was wearing a seatbelt at the time of the accident. She complained of severe abdominal pain that was primarily localized to the lower abdomen.

**Figure 1:**
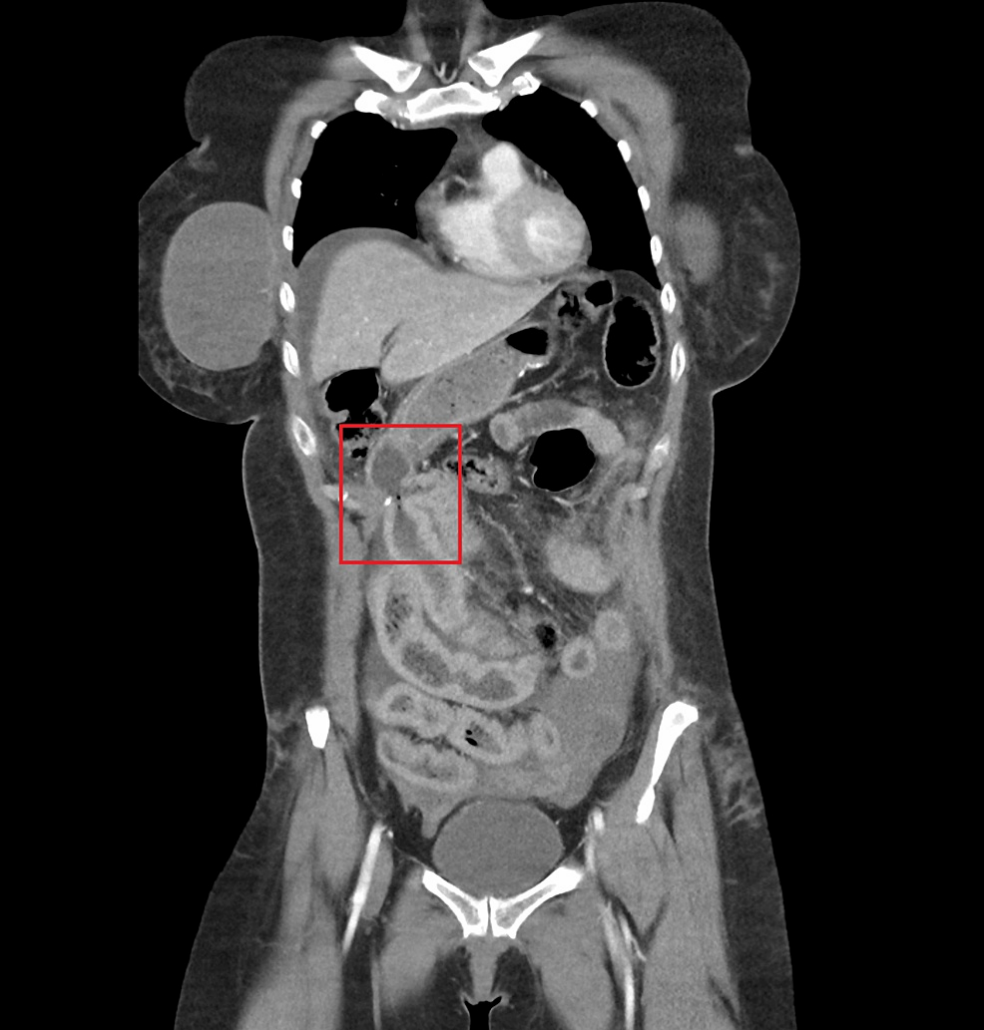
CT abdomen and pelvis scan showing SADI-S gastro-ileostomy CT: computed tomography, SADI-S: single anastomosis duodenal-ileal bypass with sleeve

**Figure 2:**
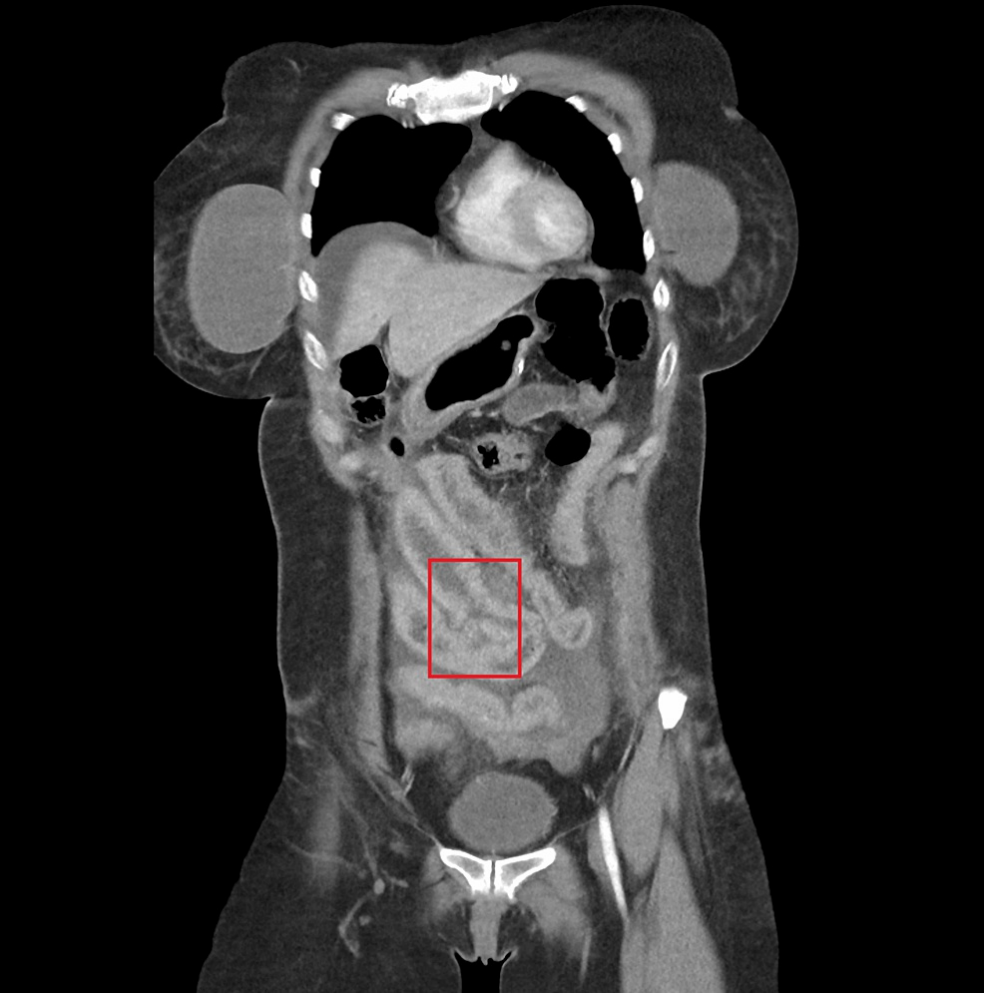
CT abdomen and pelvis showing SADI-S efferent loop wall disruption CT: computed tomography, SADI-S: single anastomosis duodenal-ileal bypass with sleeve

Her trauma survey revealed diffuse abdominal tenderness with guarding upon palpation, a left-hand abrasion, and left pretibial region ecchymosis. Abrasions of the lower abdominal wall and hips were consistent with a seatbelt injury. Her heart rate was normal upon presentation but increased to 110 after 20 minutes. She was hypertensive with a blood pressure of 159/115. Her temperature, respiratory rate, and oxygen saturation were normal. Her body mass index was 31.2 kg/m^2^. Laboratory work was significant for leukocytosis (13.5 x 10^9^/L), a hemoglobin of 14.4 g/dL, and a preoperative albumin of 4.3. X-ray imaging revealed a left anterior condyle fracture and a right anterior calcaneal avulsion fracture. A focused assessment with sonography in trauma exam was not performed. A computed tomography (CT) scan of the abdomen and pelvis revealed arterial extravasation of a gastroduodenal artery branch, venous extravasation of inferior mesenteric artery branches in the pelvis, moderate hemoperitoneum, small and large bowel contusions in the mid and lower abdomen, lower anterior abdominal wall soft tissue seatbelt contusions, and small Morel-Lavallée subcutaneous degloving injuries over the anterior hips bilaterally. Of note, the surgeon, who had expertise in minimally invasive bariatric surgery, recognized the anatomical changes associated with SADI-S on imaging (Figure 1). Upon review of imaging, disruption of the SADI-S efferent loop was noted (Figure 2).

The patient was promptly taken to the operating room for exploratory laparotomy. She received cefoxitin preoperatively. A midline incision was made through the skin and subcutaneous tissue down to the linea alba. The linea alba was incised and the peritoneal cavity was entered. Upon entry, copious bile and a small amount of blood were encountered. There were no intraabdominal adhesions from the prior surgery. After suctioning the fluid, the small bowel was run and found to be arranged consistently with a SADI-S in an antecolic position. A 5 cm perforation was identified about 20 cm distal to the duodenal-ileal anastomosis on the efferent limb. The tissue edges were damaged to a degree that made repair of this large defect unsuitable, so the segment was resected and bowel continuity restored with an isoperistaltic anastomosis. A separate 10 cm defect in the mesentery of the ileum was primarily repaired after hemostasis as it was not compromising the viability of the corresponding bowel segment. Two 19 French Jackson-Pratt drains (CardinalHealth, Dublin, OH, USA) were placed in the Morel-Lavallée lesions of the anterior abdominal wall.

She received 24 hours of cefoxitin for postoperative surgical site infection prophylaxis. The patient had an appropriate return of bowel function on postoperative day 2. She was given high-protein nutrition shakes for each meal once she was tolerating her diet. She developed a subcutaneous surgical site infection of the midline laparotomy incision for which the incision was opened and treated with a wound vacuum device. She was discharged to a skilled nursing facility.

## Discussion

Patients with prior SADI-S suffering from blunt abdominal seat belt trauma pose unique challenges. SADI-S is a relatively new and uncommon bariatric procedure. As a result, the anatomical alterations that the procedure entails are less likely to be familiar and promptly recognized in the setting of traumatic abdominal injuries. Heightened vigilance to detect injury and thoughtful intraoperative decision-making that avoids complications of bariatric surgery is important in this patient population.

CT and upper endoscopy have been used to delineate normal anatomy and postoperative complications of bariatric surgery [10]. However, CT can be challenging in diagnosing complications of bariatric surgery such as internal hernia in patients with Roux-en-Y gastric bypass [11]. Using CT, the sensitivity for diagnosing internal hernia in a patient with Roux-en-Y gastric bypass is 82% [12]. In addition, a blunt trauma patient with a seat belt sign may not always have a hollow viscus injury, though is more likely if there is the presence of intra-abdominal free fluid [13]. As a result of the obvious signs of peritonitis, our trauma patient was taken emergently to the operating room. Given the challenge of CT interpretation in bariatric patients and the unreliability of detecting hollow viscus injury in patients with a seat belt sign, it is essential to have a high index of suspicion of hollow viscus injury when treating trauma patients with previous bariatric surgery.

Biliopancreatic diversion patients with or without duodenal switch are at risk for hypoalbuminemia, anemia, and nutritional deficiencies [14]. Taking this into consideration, intraoperative decisions must be made to preserve common channel length or alter anatomy that yields a lower likelihood of malnutrition postoperatively. In this case, the small bowel was examined thoroughly to determine that the mesenteric defect could be repaired despite it being large, rather than performing a small bowel resection.

Prompt diagnosis and surgical intervention are crucial to prevent complications such as peritonitis and sepsis. Intraoperatively decisions must be made to ensure an optimal postoperative outcome. Close surveillance and patient education regarding the risks of abdominal trauma following bariatric surgery are imperative.

## Conclusions

This case highlights the importance of considering gastrointestinal injuries in patients who have undergone bariatric surgery and present with acute abdominal pain following trauma. Prompt recognition and management of such injuries and familiarity with the anatomical alterations associated with the various bariatric procedures are essential to optimize patient outcomes and prevent potential complications.
